# Mandibular small cell osteosarcoma: a case report and review of literature

**DOI:** 10.1186/s43046-023-00191-2

**Published:** 2023-09-18

**Authors:** Hatem Wael Amer, Hana’a Hezam Algadi, Shyma’a Ahmed Hamza

**Affiliations:** 1 Oral and Maxillofacial Pathology Department, Faculty of Dentistry, Cairo University, Cairo, Egypt; 2 Oral and Maxillofacial Pathology Department, Faculty of Dentistry, Hodeidah University, Hodeidah, Yemen; 3 Oral & Maxillofacial Pathology Department, Faculty of Dentistry, Newgiza University, Cairo, Egypt

**Keywords:** Small cell osteosarcoma, Osteosarcoma, Bone tumors, Mandibular osteosarcoma, Jaws neoplasms, Case report

## Abstract

**Background:**

Small cell osteosarcoma is an extremely rare histopathological variant of conventional osteosarcoma. Due to nonspecific symptoms, most osteosarcomas of the jaws are misdiagnosed as periapical abscesses and mistreated by teeth extraction and drainage.

**Case presentation:**

We report, to our knowledge, the seventh case of small cell osteosarcoma in gnathic sites affecting the mandible of an old female with history of a large painful swelling related to the right mandibular molar area for 2 months. Cone-beam computed tomography scan showed an osteolytic lesion related to the lower molar area with involvement of the inferior alveolar nerve. An incisional biopsy was taken, and after histopathological examination and immunohistochemical staining, a diagnosis of small cell osteosarcoma was reached. Hemi-mandibulectomy was performed by a maxillofacial surgeon. No clinical evidence for recurrence was noted until manuscript writing.

**Conclusion:**

Accurate diagnosis is very important, and general practitioners should be aware of this entity considering that small cell osteosarcoma has a poor prognosis when compared to conventional osteosarcoma.

## Introduction

Small cell osteosarcoma(SCOS) is a histopathological variant of conventional osteosarcoma (COS) and is extremely rare accounting for about 1 to 1.5% of all COSs [1]. It was first described in 1979 [2] and has a similar distribution as COS but is more frequently seen in the diaphysis of long bones (up to 15%). Also, it was reported to have a slightly worse prognosis than COS [3].

The clinical presentation of SCOS is not specific and is usually similar to COS, manifested most commonly as a swelling. The presence of pain, paresthesia, and ulcerations is less common symptoms [4]. The radiographical examination usually shows an osteolytic, sclerotic, or mixed radiolucent-radio-opaque lesion. Under the microscope, sheets of round cells producing an osteoid matrix can be seen.

Jaw bones are the fourth most common site for COS (particularly the mandible), accounting for approximately 6% of cases [5]. Dentists are usually the first to diagnose osteosarcomas of the jaws approximately in 45% of cases. This is due to the nonspecific symptoms, as two-thirds of the cases are misdiagnosed as periapical abscesses and mistreated by teeth extraction, drainage, and/or antibiotics [6].

Epidemiologically, SCOS are more commonly seen in the 3rd decade [7]. However, in this case report, we present the seventh case to our knowledge in gnathic sites affecting the mandible of an old female with a review of the existing literature.

## Case report

**Fig. 1:**
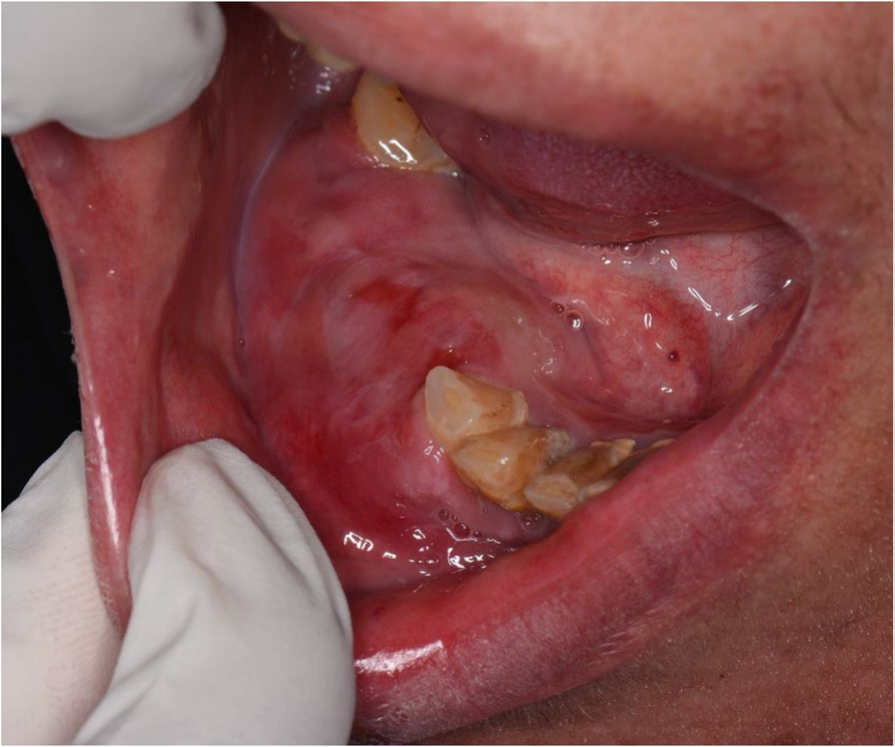
Preoperative clinical picture showing a large swelling causing buccal and lingual cortical plate expansion with nonhealing mucosa due to previous incisions

A 62-year-old female Egyptian patient attended to the outpatient clinic of the Oral and Maxillofacial Surgery Department, Cairo University in October 2022, complaining of a large painful swelling related to the right mandibular molar area (Fig. 1). The patient reported to have this swelling for 2 months. The swelling was initially diagnosed as a periapical abscess and was mistreated by teeth extractions, multiple incisions, and suturing by the general practitioner. The patient was in good physical health status, without any known health condition or any genetic disorder detected, also with no history of irradiation.

**Fig. 2:**
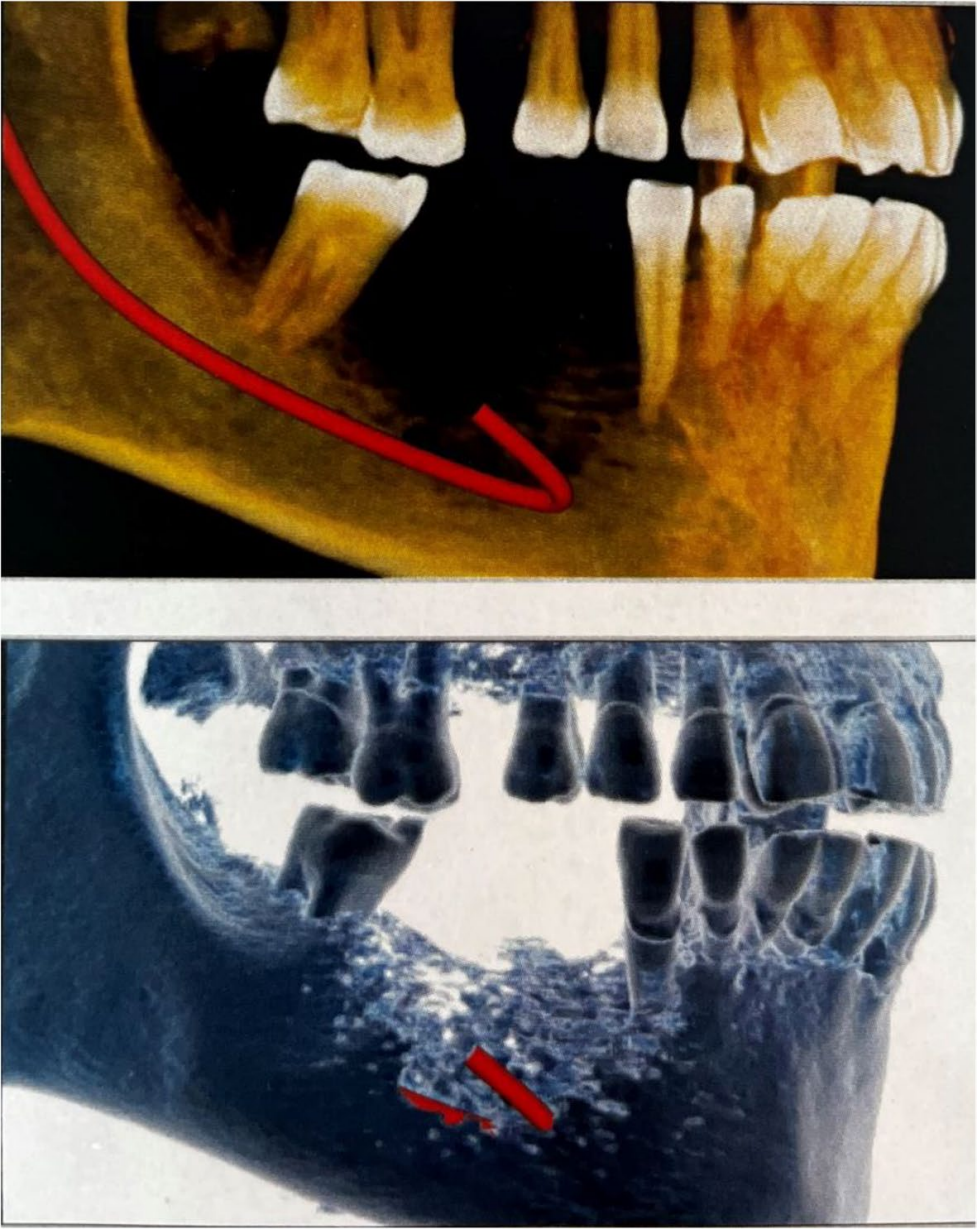
**A** CBCT image showing large radiolucent lesion related to the lower right molar area with inferior alveolar nerve involvement. **B** Negative image showing trabecular bone infiltration by tumor cells

Intraoral examination revealed expansion of the buccal and lingual cortical plates related to the lower molars area with nonhealing mucosa due to the previous incisions**.** On palpation, a firm bony mass was felt. No fluctuation of mucosa or crackling was detected. There was no clinical evidence of cervical lymphadenopathy at the time of presentation. Cone-beam computed tomography scan showed a large radiolucency with indefinite margins related to the lower right molar area, with involvement of the inferior alveolar nerve (Fig. 2). PET scan did not reveal any evidence of distant metastasis.

**Fig. 3:**
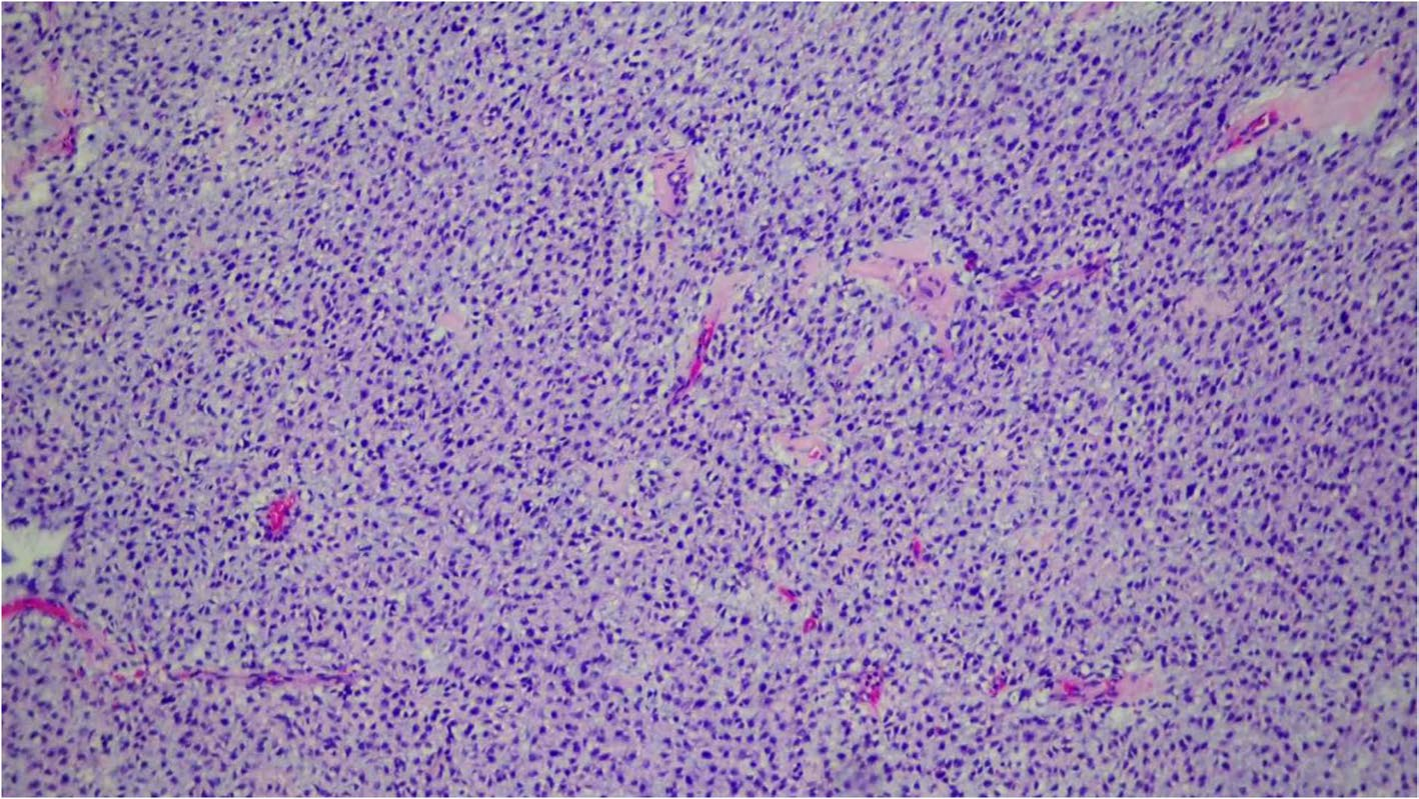
Histopathological findings of the tumor. Sheets of small round cells with minimal osteoid deposition [hematoxylin–eosin (H–E) stain, × 200]

**Fig. 4:**
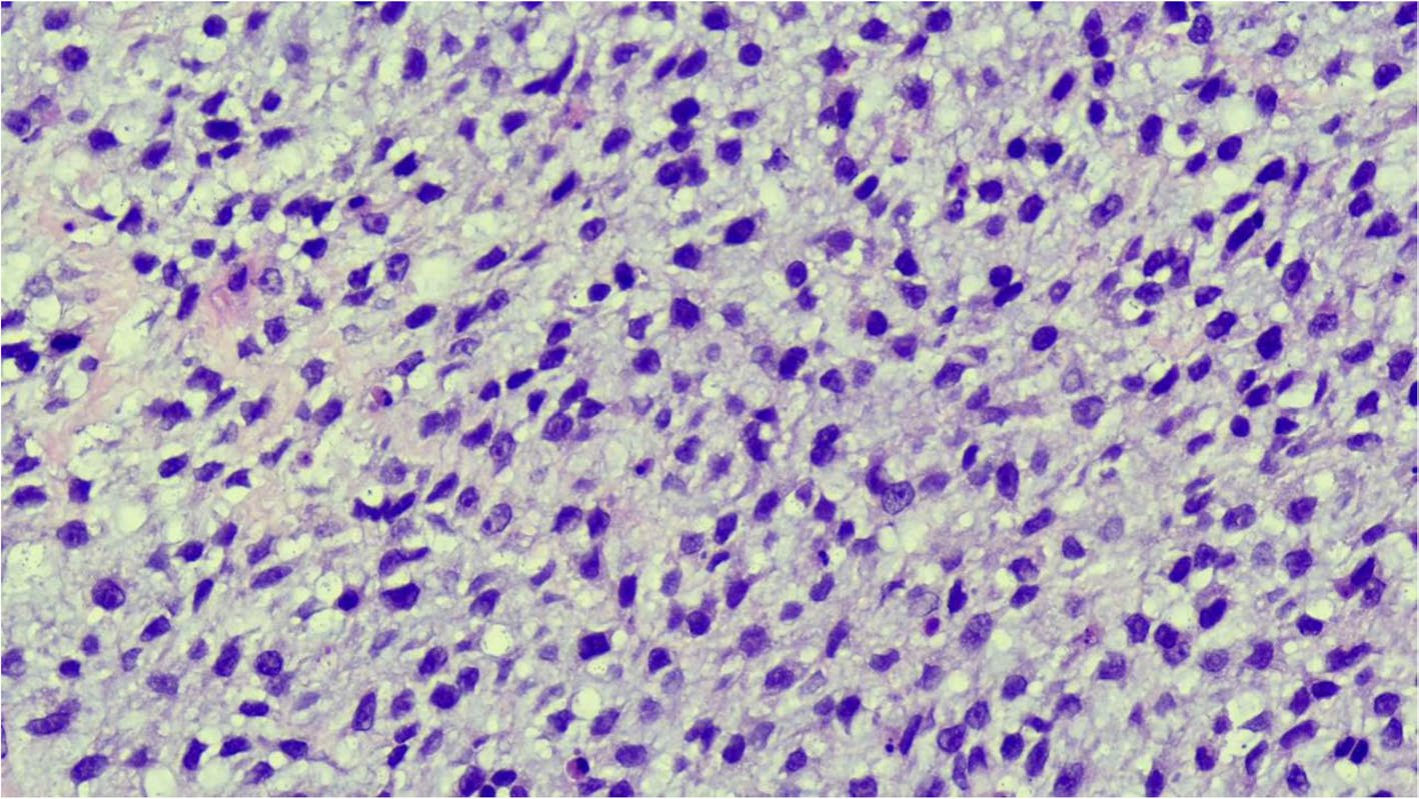
Histopathological findings of the tumor. Tumor small round cells having hyperchromatic, round nuclei, and scant cytoplasm [hematoxylin–eosin (H–E) stain, × 200]

An incisional biopsy was performed. Histopathological examination of hematoxylin and eosin-stained sections revealed sheets of round cells with an indistinct cellular outline. The cells had round-to-oval hyperchromatic nuclei, scanty cytoplasm, and distinct nuclear boundaries (Fig. 3). The malignant stroma had minimal osteoid deposition (Fig. 4).

**Fig. 5:**
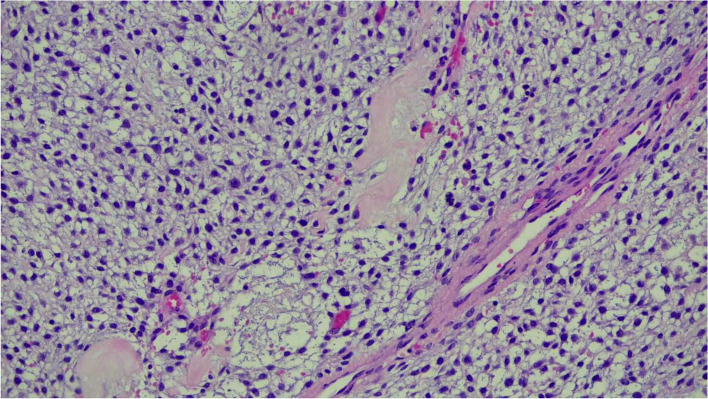
Histopathological findings of the tumor. Small round cells and evident osteoid formation [hematoxylin–eosin (H–E) stain × 200]

**Fig. 6:**
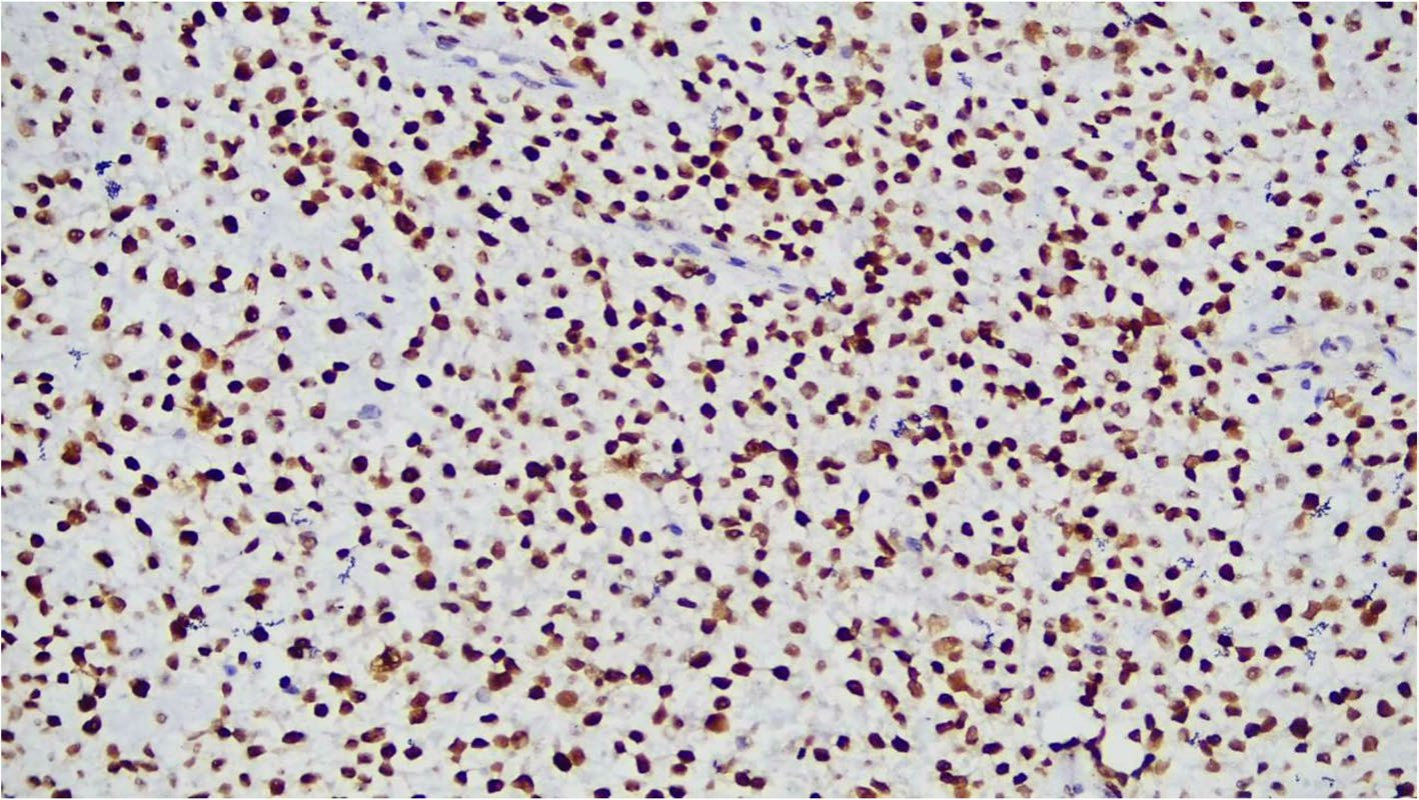
Immunohistochemical staining of tumor cells showing diffuse positive nuclear staining for SATB2 marker × 200

The diffuse-positive nuclear immunohistochemical staining (IHC) of special AT-rich sequence-binding protein 2 (SATB2) marker confirmed the diagnosis of SCOS (Figs. 5 and 6). It was performed to exclude other small round cell tumors that occur in old age [8] such as non-Hodgkin lymphoma, small cell neuroendocrine carcinomas, poorly differentiated synovial sarcoma, and metastatic small cell carcinoma [9, 10].

Based on the combination of IHC evidence of osteoblastic differentiation of the tumor cells along with the presence of small round cells-producing bone matrix led to the final diagnosis of SCOS. Hemi-mandibulectomy with a 10-mm clear surgical margin was performed by maxillofacial surgeon. The reconstruction plate was used to join the condyle with the remaining part of mandible. After 6 months, the patient had undergone free fibula microvascular flap reconstruction. No adjuvant chemotherapy and/or radiotherapy was done. The histopathological examination and IHC staining results obtained from the received specimen were similar to the results of the earlier incisional biopsy. Follow-up of the patient showed no clinical evidence of recurrence until manuscript writing.

## Discussion

In the recent 5th edition of the WHO classification of soft tissue and bone tumors, small cell osteosarcoma was merged under COS as a histopathologic variant [5]. Before that, it was considered as a stand-alone subtype of osteosarcoma for a long time. It is a rare variant, comprising about 1.5% of all osteosarcomas, first described by Sim et al. [11] as a distinct entity osteosarcoma with small cells simulating Ewing’s sarcoma.

The documented cases of SCCO were reported in several sites of the skeleton, such as pelvis and humerus [7]. However, to the extent of our knowledge, in gnathic sites (maxillary and mandibular jaws), there are only 6 reported cases in the English language literature (Table 1), whereas 5 cases were reported in the mandible and only one in the maxilla. We report the seventh case of gnathic sites affecting the mandible of a 62-year-old female. The reported median age of diagnosis was 26.7 affecting both sexes almost equally [6, 12–16].

**Table 1:** Providing a clinicopathological summary of the seven reported cases of the jaws, between 1984 and 2022

Case no	Author(s)/year	Sex/age	Location	Radiology	Treatment	Clinical outcome
1	[12] Giangaspero, F. et al./1984	F/8	Anterior mandible	Bone resorption	SR/chemotherapy	NR
2	[14] Kim, Y. et al./1999	M/25	Right retromolar triangle	Focal disruption of the inner cortex of the mandible	SR	NR
3	[16] Sethi, A. T. et al./2010	M/18	Near the left angle of the mandible	Ill-defined radiolucency	SR	NR
4	[23] Uma, K. E. T. et al./2011	F/28	Left mandibular angle	Ill-defined radiolucency	SR/chemotherapy	NR/M
5	[13] Harazono, Y. et al./2015	F/26	Left mandible	Destruction in the left lingual cortical bone	SR, neck dissection/chemotherapy	NR/M
6	[15] Selvakumar et al./2017	M/37	Left maxilla	NR	Complete maxillectomy	Local recurrence and distant metastases
7	The present case/2022	F/62	Right mandible	Bone resorption	SR	To date no recurrence

*F* female, *M* male, *NR* not recorded, *NR/M* no evidence of recurrence or metastasis, *SR* surgical resection

To date, there is no specific molecular genetic alteration for SCOS. However, in some documented cases, there were many patients who had either Ewing sarcoma breakpoint region 1 (EWSR1) or BCOR that encodes the BCL-6 corepressor protein rearrangements reported [17–20]. Interestingly, Noguera et al. reported one case of translocation (11; 22) [21]. In addition, by genomic sequencing of the breakpoint between EWSR1 and CREB3L1, Debelenko et al. confirmed the chimeric fusion gene transcripts [22].

All the documented cases of SCOS shared the same histopathological criteria which are sheets of small round cells with indistinct cellular outlines separated by dense fibrous tissue. The tumor cells usually have scant eosinophilic cytoplasm with nuclei that are generally small to medium and round to oval in shape. The presence of osteoid in the stroma is considered a cornerstone for SCOS differentiation from other small round cell tumors. In our case, the diagnosis was challenging because the malignant stroma had minimal osteoid deposition arranged in a lacelike fashion.

SCOS stain positively to considerable number of IHC markers such as CD99, vimentin, osteocalcin, osteonectin, and cytokeratin [5, 16, 23]. These markers also stain several round cell tumors. The special AT-rich sequence-binding protein 2 (SATB2) is an important transcription factor for osteoblastogenesis and is considered a highly sensitive marker for osteoblast lineage differentiation. In our case, it was used to rule out the other undifferentiated round cell sarcomas such as Ewing and the so-called Ewing-like sarcoma, and rhabdomyosarcoma (these previous tumors usually occur in the pediatric age) [7,  18]. Moreover, the positive staining helped us to exclude other round cell tumors that share various degrees of similarity with SCOS like non-Hodgkin lymphoma, metastatic small cell melanoma, poorly differentiated synovial sarcoma, small cell carcinoma, neuroblastoma, and mesenchymal chondrosarcoma [7].

Surgical resection with clear margins was done for the patient, and this is considered the treatment of choice [5]. Aggressive lesions with local invasion needed adjuvant therapy like chemotherapy and radiotherapy [15]. Follow-up of our patient did not show any evidence of recurrence until writing this manuscript. It is worth mentioning that, in three of the documented cases, the recurrence was not recorded [12, 14, 16], two of the documented cases showed no evidence of recurrence [13, 23], and one case showed local recurrence in the orbit after 1 year and a half [15]. The use of SATB2 could be a possible strength point in this study, as it is considered a highly sensitive marker for osteoblast lineage differentiation. However, the absence of diagnostic molecular genetic testing and short follow-up period could be the limitation.

## Conclusion

In conclusion, SCOS is an extremely rare variant histopathological of COS of the head and neck area and has aggressive behavior with local invasion leading to difficulty in controlling the lesion and ultimately a high mortality rate. The takeaway lesson is that general practitioners must be aware of this entity that has no specific symptoms to avoid misdiagnosis and mistreatment as an abscess. The special AT-rich sequence-binding protein 2 (SATB2) IHC marker is a helpful tool to reach a proper diagnosis, especially when osteoid production is minimal to differentiate SCOS from other histopathological mimickers’ tumors. The accurate diagnosis is very important to achieve an effective curative regimen that will improve patient survival, considering that SCOS has a poor prognosis when compared to COS (2).

## Data Availability

The data that support the findings of this study are available from the corresponding author upon reasonable request.

## Abbreviations

COS: Conventional osteosarcoma
SCOS: Small-cell osteosarcoma
IHC: Immunohistochemical
SATB2: Special AT-rich sequence-binding protein 2
